# Rationale for setting up a cardio-oncology unit: our experience at Mayo Clinic

**DOI:** 10.1186/s40959-016-0014-2

**Published:** 2016-04-19

**Authors:** Sergio Barros-Gomes, Joerg Herrmann, Sharon L. Mulvagh, Amir Lerman, Grace Lin, Hector R. Villarraga

**Affiliations:** Mayo Clinic, Department of Cardiovascular Diseases, 200 First Street SW, Rochester, MN 55905 USA

**Keywords:** Cardio-oncology, Cardiotoxicity, Cardiovascular risk factors, Multidisciplinary, Cardio-oncology program, Electronic medical records

## Abstract

**Background:**

The diagnosis and management of cardiovascular complications have become a clinical concern for oncologists, cardiologists, surgeons, interventional radiologists, radiation therapy physicians, internists, nurses, pharmacists, administrators, and all the stakeholders involved in the care of cancer patients. Anticancer therapies have extended the lives of patients with cancer, but for some this benefit is attenuated by adverse cardiovascular effects.

**Methods:**

This review article aims to provide an overview of the rationale of setting up a cardio-oncology unit and reflect on our own experience establishing this service, and conclude with some fundamental aspects of consideration for evaluation and management of patients with cancer and cardiovascular diseases.

**Results:**

Cardiotoxicity can lead to congestive heart failure and cardiac death. In fact, chemotherapy-related cardiac dysfunction may carry one of the worst prognoses of all types of cardiomyopathies, and has a profound impact on morbidity and mortality in oncology patients. Other complex clinical situations involve cancer patients who might benefit from a highly curative drug in terms of cancer survival but face limitations of its administration because of concomitant cardiovascular diseases. Indeed, the balance between the benefits and risks of the cancer therapy regimen in the context of the cardiovascular status of the individual patient can sometimes be extraordinarily challenging. A subspecialty with a multidisciplinary integrative approach between oncologists, hematologists, cardiologists, among others has thus emerged to address these issues, termed cardio-oncology. Cardio-oncology addresses the spectrum of prevention, detection, monitoring and treatment of cancer patients with cardiovascular diseases, or at risk for cardiotoxicity, in a multidisciplinary manner. In this field, cardiologists assist oncologists and hematologists with cardiovascular recommendations. This can be mediated through e-consultations or face-to-face evaluations.

**Conclusion:**

Cardio-oncology is a subspecialty that assists in the overall care of cancer patients with and without cardiovascular disease in an interdisciplinary fashion. We believe that this partnership of sharing responsibilities and experiences among health-care team members can potentially decrease cancer therapeutics-related cardiovascular complications and improve clinical outcomes.

## Background

Population growth and ageing as well as improvements in early diagnosis and anticancer therapies has led to a projected nearly 19 million cancer survivors in the United States alone by the year 2024 [1–3]. As successful anticancer therapies are developed, the benefit comes with an increased number of cardiovascular complications [4–6]. In the past decades, the risk of congestive heart failure (CHF) with high cumulative dose of anthracyclines was found to be from 3 to 26 % [6–11]. With improved knowledge and reduction of the total anthracycline-dose, this cardiotoxicity risk of anthracyclines has been reduced to nearly 2-3 % over a time period that extends at least 5 years [12], but with the increased incidence and survival rates of cancer patients in an aging population that is at greater risk for complications with chemotherapy, the number of patients with cardiac complications remains high [1, 3, 5, 13, 14]. Although it has been extremely difficult to know the incidence and prevalence of chemotherapy-induced cardiotoxicity (due to limitations on the definition, the lack of reportable data regarding cardiotoxicity, and the presence of selection bias in recruiting special populations, etc) [15], this has also been outlined in a cohort of patients referred for endomyocardial biopsy that chemotherapy-induced cardiomyopathy carries one of the worst prognoses of all types of cardiomyopathies [16]. Additionally, there are other chemotherapeutic- and radiotherapeutic-related cardiovascular complications besides overt cardiac dysfunction that can negatively impact the overall outcome of cancer patients, including hypertension, ischemia, and arrhythmias [5, 17–19].

Therefore, early recognition of cancer therapy-related toxicity has become a clinical concern for hematologists, oncologists, and cardiologists [12, 20, 21]. A subspecialty that includes an integrative multidisciplinary approach to this issue has established, termed cardio-oncology [22–24]. The origins of the discipline date back late in 1960s, when cardiac dysfunction resulting from anthracyclines was first recognized as an important side effect. The field since then has arisen in few centers, and in the past years has rapidly evolved and become more a formal subspecialty with smaller units emerging within major centers. The scope of cardio-oncology includes not only the prevention, detection, monitoring and treatment of cardiovascular toxicity related to cancer therapy but also to assist in the overall care of cancer patients from cancer diagnosis into survivorship. The goal is to provide optimal care for patients with cancer and cardiovascular disease. A brief discussion of the cardiotoxicity disease spectrum is provided in the first part of this review article. We will then provide an overview about the rationale of setting up a cardio-oncology service line and our initial experience of establishing a collaborative cardio-oncology program within our practice will be presented, emphasizing important points of consideration in the cardiovascular evaluation before, during, and at completion of anticancer treatment.

### Cardiotoxicity

There are different cardiovascular manifestations related to chemotherapy. There are agents that primarily affect cardiac function (eg, doxorubicin [anthracycline], cyclophosphamide [alkylating agent], and trastuzumab [tyrosine kinase inhibitor]). In addition, there are agents that indirectly contribute to cardiac decompensation by altering preload (imatinib [VEGFi] through fluid retention), afterload (bevacizumab [VEGFi] through hypertension), and heart rate (ifosfamide [alkylating agent] through arrhythmias) and agents that cause cerebrovascular disease (5- cisplatin [alkylating agents - platinum], 5-fluorouracil [antimetabolites]) [19–25]. There is also radiotherapy that has an all-inclusive involvement of the heart (myocardium, pericardium, valves and coronary arteries) [26] and can affect extra cardiac structures such as the great vessels where accelerated atherosclerosis can occur [27]. However, a reduction in left ventricular ejection fraction (LVEF) and subsequent development of CHF has drawn most of the attention among physicians. Cardiac function impairment as a consequence of cancer therapy was first recognized in the 1960s [28], may be reversible or irreversible, and can occur acutely (at the time or within 1 week) or chronic with early (<1 year) and late onset (>1 year) after completion of chemotherapy [29, 30]. Importantly, chemotherapeutic agents are implicated in the development of myocardial ischemia, hypertension, hypertensive heart disease, or a combination, which may lead to left ventricular dysfunction [31, 32].

An operational classification model has been introduced distinguishing two types of cardiotoxicity [33]. Type I causes a direct irreversible damage to the cardiomyocyte, mainly in a dose-dependent manner [34, 35], as observed with anthracyclines [11]. Conversely, a type II cardiotoxicity pattern entails cardiac dysfunction with less prominent structural injury or irreversible cell damage since electron microscopy has shown structural changes in the animal model with trastuzumab [36, 37]. Type II cardiotoxicity does not exhibit dose dependency, is usually transient and carries a better prognosis [31].

### Rationale for a multidisciplinary approach

Cardiovascular complications from cancer therapy have become a leading cause of morbidity and mortality in cancer survivors [7, 38]. Anticancer therapies have extended the lives of patients with cancer, but for some at the cost of adverse cardiovascular events [6, 12]. Increasing age, underlying heart disease and other comorbidities are contributing factors. Moreover, a variety of cardiovascular scenarios can occur in this population. For patients with an advanced metastatic tumor, the development of heart failure compromises their quality of life and palliative care. In contrast, for patients with a high likelihood of cure, chemotherapy-induced heart failure significantly impacts their long-term outcome [12]. Additionally, we are often confronted with challenging decisions on drug therapies beforehand based on the curative benefit on the one hand and cardiotoxicity risk on the other hand in patients with significant cardiovascular risk factors. These challenges have advocated the compelling need for the multidisciplinary integrative approach of cardio-oncology [22, 39–41]. Cardio-oncology aims not only to detect and manage cardiotoxicity but also to assist in the overall care of cancer patients with and without heart disease in an interdisciplinary manner that ranges from the initial assessment of cardiovascular diseases and cardiotoxic risks to survivorship and long-term follow-up.

The multidisciplinary role becomes even more important as cardiotoxicities are identified at earlier stages of cancer treatment than they used to be. However, while much progress has been made in early detection and management of toxicities, there has been less progress in the understanding of short- and long-term outcomes of cancer therapies and intervention efforts. The cardiologist needs to know the goal of the oncology treatment, whether this treatment is curative or palliative, and the potential anticipated benefit of anticancer therapy to further assist the oncologist/hematologist [40]. Mutual understanding and the communication between the cardiologist and oncologist/hematologist needs is paramount for risk stratification and decisions on the therapeutic window for any given therapy. Indeed, there is a critical balance between potential benefits and risks of different chemotherapeutic regimens and the need of the patients.

**Fig. 1:**
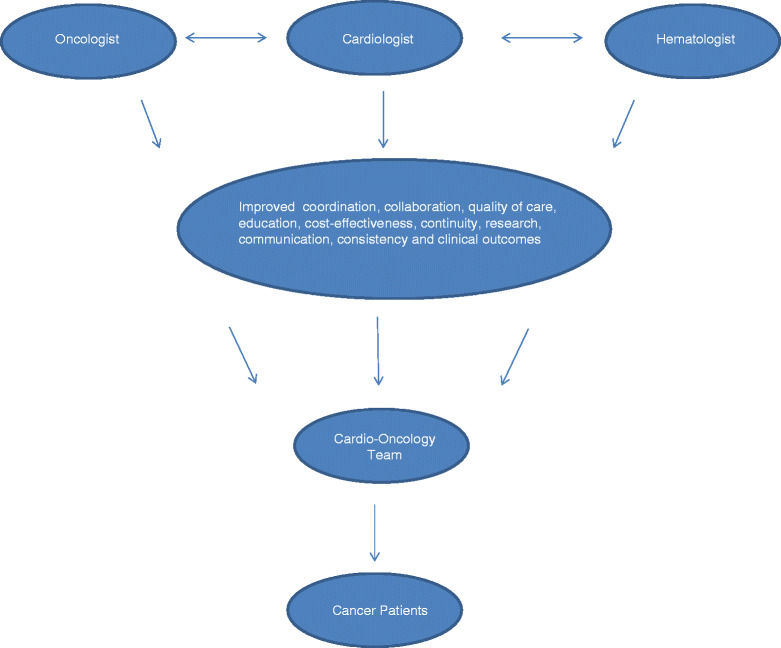
Cardio-Oncology Multisciplinary Team. The integrative approach increases the coordination, communication and collaboration between health-care members and improves the overall care of cancer patients

Accordingly, one of the main goals of cardio-oncology is to promote open discussions between team members in order to share expertise and responsibilities. Integrating expertise from all health-care members provides a constant high-level standard of care. It is our expectation that this discipline will reduce the incidence of cardiotoxicity, improve development of new anticancer drugs, and positively impact overall patient care. The integration of all involved health-care providers and patients is a key element to improving the quality of care [41, 42] Fig. 1.

### Setting up the cardio-oncology practice

Mayo Clinic has established a Cardio-oncology Clinic to improve the overall acute and long-term outcome of cancer patients. This subspecialty was initially created: 1) to facilitate the diagnosis, monitoring and therapy of cancer treatment related cardiovascular complications; 2) to evaluate baseline cardiovascular risks prior to cancer treatment and implement strategies for risk reduction of developing cardiovascular complications; and 3) to assist the patient with cardiovascular care through long-term follow up. The multidisciplinary team consists of cardiologists with additional expertise in prevention, heart failure, vascular disease, and cardiovascular imaging. It also encompasses oncologists, hematologists, internists, nurses, pharmacists, and all others involved in the care of cancer patients. As previously mentioned, the interdisciplinary communication and coordination is crucial to the operational functionality of the cardio-oncology practice.

At our institution, the cardio-oncology practice was initially established through the internal electronic referral management system (“e-consults”). E-consultations are electronic-based consultations where the specialist the “e-consultant” answers questions and provides advice about patient care. The referring provider generates a question to the consulting specialist with the appropriate clinical material and the e-consultant specialist answers it through the electronic medical record. There is no patient verbal contact, only medical assistance through the patient medical records [43–45]. These electronic-based consultations are provided by a cardiologist of the cardio-oncology team in response to specific questions. These types of consultations emerged as a mechanism to provide efficient clinical care in a timely manner. In cardio-oncology, for instance, this method enables cardiologists to further assist oncologists and hematologists to assess risk factors and manage existing cardiovascular diseases. The implementation of e-consultation is only feasible in the presence of an electronic medical record (EMR), which is another crucial element that avoids the fragmentation of data between patients and providers [46]. The EMR system provides a continuum of communication and clarification of information, wherein physicians have easy access to patient’s charts, laboratories, and procedures (ie, ECG, echocardiogram, etc). This integration between two systems (e-consult and EMR) delivers a high-quality coordinated care that potentially avoids the time and wait of a visit between the patient and specialist.

With the growth of the cardio-oncology practice as well as based on the explicit demand of cancer patients or their providers, face-to-face consultations were added and became the main mode of service. It is recognized that the cardio-oncology patient has a high diagnostic and treatment complexity, prompting more direct interactions with the individual patient. Each cardio-oncology service faces particular challenges that are associated with the size of the hospital, the volume of patients, and the scope of cancer treatment. Joint meetings with oncology and hematology counterparts were held to define this practice and its logistics. This included discussions on criteria for referral as an e-consult or face-to-face consultations, standards of pre-orders of tests, and best possible location and timing of a full clinic. Other topics of discussion included educational seminars and conferences for patients and health staff, the establishment of a database for future research, and the development and integration of a cardio-oncology-specific fellowship program as shown in Table 1 [47, 48].

**Table 1:** Setting up a cardio-oncology clinic

**Define practice and logistic**	Recognize gaps and priorities in cardio-oncology Joint meetings with cardiologists, oncologists, hematologists, nurses and nurse practitioners, pharmacists, nutritionists, rehabilitation services, palliative care, and social services Discuss criteria for referral consultations, standards of pre-orders of tests (biomarkers and strain), location and timing of a full clinic, integration of services, education and training of staffs
**Implement a coordinated service**	Exchange patient information with the counterparts, allow a flexible scheduling system to accommodate a multidisciplinary team, ensure an updated medications list (cardiac and oncologic regimens)
**Health staff education**	Teaching material on cardio-oncology, updates, educational seminars, symposium and conferences Provide awareness of the cardio-oncology program
**Patient education**	Patient booklet, educational website, seminars, symposium, and community events
**Standardization of care**	Create algorithms, cardio-oncology group meetings, joint educational sessions with oncology, hematology and cardiology
**Research**	Conduct lab-based experimental studies, apply for funding and awards, registry expansion (clinical data and bio bank), and create clinical and laboratory facilities with new techniques (biomarkers and strain)
**Administrative**	Every other month meetings with updates and outcomes Establish targets and goals

Bold data emphasize the most important content from the Table

**Fig. 2:**
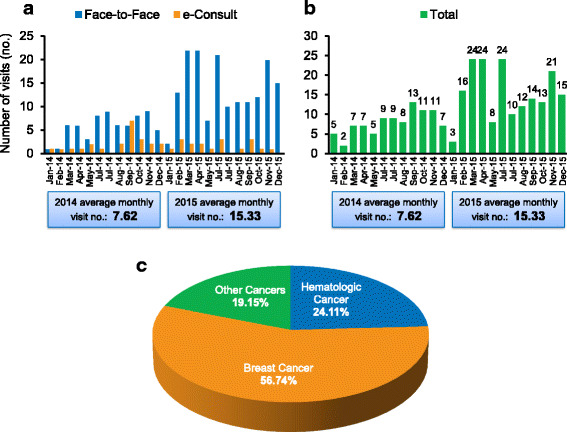
Cardio-Oncology Visits and Referral Source. **a** and **b** Bar charts showing the average monthly visits in 2014 and 2015. **c** Cardio-Oncology Referral Source. Pie chart displaying the proportion of type of cancers referred to the Cardio-Oncology Clinic

A formal Cardio-Oncology Clinic was then started and had a significant growth over the past 2 years. The monthly number of visits has increased by 101.3 % since 2014 to a 2015 monthly average of 15.33 (Fig. 2a-b). The ratio between new and old patients is 3:1. Breast cancer was the most frequent (56.7 %), followed by hematological cancers (24.11 %) (Fig. 2c). These are usually complex patients that demand a complex care, which require medical assistants, nurse and nurse practitioners, and physician extenders (physician assistant, fellows and internists). Some of these patients are in the intermediate or high risk category for coronary artery disease, cerebrovascular disease or heart failure. This management also includes coordinated home health monitoring of blood pressure, cholesterol levels, diuresis, medications adjustments, and evaluation of new symptoms through e-mail or phone calls. Nurse, nurse practitioners, and physician extenders are able to see more stable or return patients, therefore allowing the cardio-oncologist more time to see more complex patients.

The Clinic has slowly expanded to avoid miscommunication in the coordination of patient care, since this is a multidisciplinary team and all efforts have been focused on avoiding errors due to a lack of adequate communication among team-members. This is accomplished by an integrated electronic medical record that ensures that all the clinical impressions, reports and plans are available to all the care team.

Our goals for this current year are to establish Cardio-Oncology group meetings every other month and standardization of care (Mayo algorithms); expansion of care (increasing referral and patient volume as well as further integration into survivorship and rehabilitation); joint educational sessions with oncology, hematology and radiation therapy, applications for institutional, extramural, and industry grants; initiation of new lab-based experimental studies; continuation of ongoing experimental collaborations; continuation of two cardiovascular prospective awards; registry expansion (clinical data, bio bank) with research nurse support; and a Mayo Clinic Cardio-Oncology Symposium. We hope that we and other new cardio-oncology programs may bring improvements in clinical outcomes and may contribute to health and well-being in patients with cancer.

### Baseline and monitoring evaluation of oncology patients

From a clinical practice standpoint, prediction of the risk of cardiotoxicity has a very high priority as it allows for better allocation and individualization of therapy. A formal recommendation has been recently proposed from the ASE Expert Consensus Group [20], wherein cancer therapeutics-related cardiac dysfunction (CTRCD) is defined as a decrease in LVEF of > 10 percentage points, to a value < 53 %. Mayo Clinic established a standardized approach based on this consensus and our own experience.

**Fig. 3:**
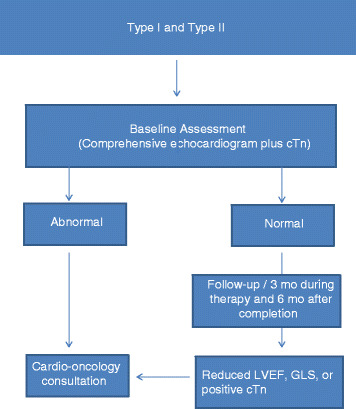
Type I and Type II cardiotoxicity. Baseline and serial evaluation in patients receiving combined therapies with drugs with both type I and type II toxicity risk. Echocardiogram and cardiac biomarkers are performed during baseline. For abnormal baseline screening, we suggest cardio-oncology consultation. For normal baseline screening, we suggest serial monitoring with echocardiogram and biomarkers every 3 months during therapy and 6 months after completion of treatment. F/U indicates follow-up; GLS, global longitudinal strain; LVEF, left ventricular ejection fraction; cTn, serum cardiac troponin

Accordingly, it is our practice that patients at risk of type I cardiotoxicity undergo a comprehensive echocardiographic evaluation and biomarkers screening at baseline, completion of therapy and 6 months later. As per consensus [20], we recommend echocardiographic evaluation with strain (global longitudinal strain [GLS] using two-dimensional speckle-tracking echocardiography [2D-STE]) and serum cardiac troponin (cTn) after a cumulative dose of 240 mg/m^2^ has been achieved and additional evaluations before each additional 50 mg/m^2^ of anthracycline. In those at risk of type II cardiotoxicity, echocardiograms and biomarkers are performed every 3 months during treatment. For those receiving combined therapies with drugs with both type I and type II toxicity risk, echocardiograms and biomarkers are performed every 3 months during therapy and at 6 months after completion of treatment [20]. Figure 3 illustrates our baseline and serial monitoring of the oncology patient with drug therapy at risk of developing Type I and Type I toxicity [20].

It is important to recognize that this follow-up may vary according to risk factors and individual patient characteristics and/or genetic susceptibility. For patients at increased cardiac risk, a more aggressive cardiac monitoring regimen should be considered. Thus, the recommended cardio-oncology consultation strategy includes a detailed medical history (eg, with emphasis on any heart disease and a comprehensive echocardiogram with strain imaging), type of anticancer therapy to be initiated (including planned cumulative dose and rate of administration), and the presence of risk factors. For risk assessment, a thorough history and physical examination is obtained, including age, cardiovascular risk factors, and history of prior exposure to agents and/or radiotherapy. In combination with information on planned therapies, the overall perceived risk can be illustrated by a score value [21]. The elements are in agreement with a recent meta-analysis that integrates specific risk factors for cardiotoxicity [49], age (<15 or > 65 year), female, prior cardiomyopathy, ischemic heart disease, hypertension, diabetes, use of anthracycline, and chest radiation were associated with increased risk. Based on that, a baseline risk assessment should be performed and a cardiotoxicity risk score can be calculated as shown in Table 2 [21]. Hemodynamic parameters, such as volume status, heart rate, and blood pressure should be optimized before initiating treatment.

**Table 2:** Risk assessment and monitoring associated with left ventricular dysfunction

**Patient-related risk factors**	**Medication-related risk factor** ^**a**^
**1 point for each risk factor present**	***High (risk score 4):*** Anthracyclines, Trastuzumab, Ifosfamide, Cyclophosphamide, Clofarabine
Age (bimodal distribution): <15 or > 65 years Female Hypertension Diabetes Mellitus Atherosclerosis (coronary artery disease, cerebrovascular disease, peripheral artery disease) Preexisting heart disease or heart failure Prior anthracycline Prior radiation therapy to the chest	***Intermediate (risk score 2):*** Docetaxel, Pertuzumab, Sunitinib, Sorafenib
	***Low (risk score 1):*** Bevacizumab, Imatinib, Lapatinib, Dasatinib
	***Rare (risk score 0):*** Etoposide, Rituximab, Thalidomide
***Cardiotoxicity Risk Score (CRS)*** Medication-related risk score + number of patient-related risk factors = *CRS > 6*: very high; *CRS 5-6*: high; *CRS 3-4*: intermediate; *CRS 1-2*: low; *CRS 0*: very low	
**Mayo Clinic monitoring recommendations**	
***Very high risk:*** Echocardiogram with GLS before every (other) cycle, end, 3-6 months and 1 year. Optional ECG, cTn with echocardiogram during chemotherapy	
***High risk:*** Echocardiogram with GLS every 3 cycles, end, 3-6 months and 1 year after treatment. Optional ECG, cTn with echocardiogram during chemotherapy	
***Intermediate risk:*** Echocardiogram with GLS, mid-term, end and 3-6 after treatment. Optional ECG, cTn mid-term of chemotherapy	
***Low risk***: Optional echocardiogram with GLS and/or ECG. cTn at the end of treatment	
***Very low risk***: None	

Risk assessment, cardiotoxicity risk score at the time of baseline assessment, and monitoring for patients undergoing anticancer therapy. ECG indicates electrocardiogram; GLS, global longitudinal strain; cTn, serum cardiac troponin. From Herrmann J et al. [21], with permission. ^a^Medication-related risk factor (1-4) was based on the risk for a decline or dysfunction in the ventricular function. Bold to emphasize the most important components

**Fig. 4:**
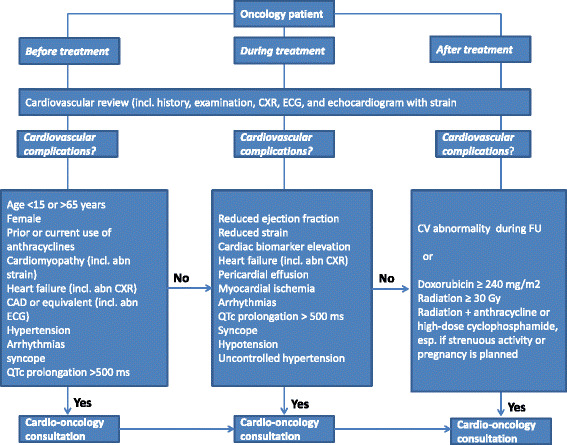
Cardio-Oncology General Practice. Figure depicts our general cardio-oncology practice before, during and after chemo and/or radiation therapy (from Herrmann J et al. [21], with permission). abn indicates abnormal; CAD coronary artery disease; CXR, chest x-ray; ECG, electrocardiogram; QTc, corrected QT

As an important part of baseline clinical work-up prior to cancer treatment, we recommend chest x-ray, ECG, biomarkers (cTn and/or brain natriuretic peptide [BNP]), and echocardiography with strain imaging in all patients who are to undergo treatment regimens that bear cardiotoxicity risk (Fig. 4). Abnormal echocardiographic examination (reduced LVEF or GLS obtained by 2D-STE) and/or biomarkers (elevated cTn or BNP) require a cardio-oncology consultation. Quantitative assessment of LVEF using 2D Simpson’s biplane, and/or 3D echocardiography, with or without contrast (as needed for optimization of endocardial border definition) are clinically indicated. It is well known that LVEF is not a very sensitive index to detect subtle changes in myocardial contractility [20, 50]. More sensitive indices, such as GLS by 2D-STE, can detect early changes in intrinsic myocardial function and thus predict CTRCD. We have recently shown in patients with lymphoma [51], breast cancer (mainly anthracycline based chemotherapy) [52], and those undergoing treatment with VEGFi [53] that GLS measured by 2D-STE can detect early cardiac damage before a decrease in LVEF is identified. The method has been widely used to monitor cancer patients undergoing chemotherapy [20, 50–56].

Also, cardiac biomarkers have been shown to have incremental value in the detection of CTRCD [54–60]. cTn in particular was able to predict CTRCD in a very early phase of treatment [57, 58]. Cardinale et al. demonstrated that patients without cTn elevation after chemotherapy completion have a good prognosis whereas persistence of positive values for 1 month is associated with a higher incidence of cardiovascular events (87 %) [61]. In particular, cTn can be used to identify lower risk patients (higher negative predictive value). However, its predictive value is not superior and possibly not additive to that obtained with strain imaging (ie, GLS by 2D-STE) [55, 62].

Although much progress has been made, we believe we do not know the best method of monitoring these patients, how long they should be monitored, or the ways that these new techniques (strain imaging and biomarkers) will impact on survivorship. Anticancer therapies have brought hope and cure and extended the lives of patients with cancer, but for some these remarkable advances are attenuated by adverse cardiovascular effects. Mutual understanding and open discussions between team members in order to share expertise and responsibilities are required to achieve the best outcome for the patient.

## Conclusion

A subspecialty with a multi-disciplinary integrative approach has emerged termed cardio-oncology. Cardio-oncology has the scope of diagnosing, preventing and treating patients with cancer and cardiovascular diseases. The discipline assists in the overall care of cancer patients from cancer diagnosis into survivorship. In this field, cardiologists assist oncologists and hematologists to further assess risk factors and manage existing or developing cardiovascular diseases. This partnership of shared responsibilities among multi-disciplinary professionals is a key element in improving the quality of care for cancer patients. This can be mediated through e-consultations or face-to-face evaluations and reported in an electronic medical record for better communication with all stakeholders involved in the care of the cancer patient. It is anticipated that this multidisciplinary approach will have an impact in decreasing cancer therapeutics-related cardiac dysfunction and improve patient outcomes.

## Abbreviations

2D-STE: two-dimensional speckle-tracking echocardiography
BNP: brain natriuretic peptide
CHF: congestive heart failure
cTn: serum cardiac troponin
CTRCD: cancer therapeutics-related cardiac dysfunction
ECG: electrocardiogram
e-consult: electronic referral management system
EMR: electronic medical records
GLS: global longitudinal strain
LVEF: left ventricular ejection fraction
VEGFi: vascular endothelial growth factor inhibitor
